# Immune Dysregulation in HIV-TB Co-Infection: Role of Cytokines and T Cell Biomarkers—A Narrative Review

**DOI:** 10.3390/pathogens15010051

**Published:** 2026-01-03

**Authors:** Catherine Keiko Gunawan, Anton Sumarpo, Agnes Rengga Indrati

**Affiliations:** 1 Medical Profession Study Program, Faculty of Medicine, Maranatha Christian University, Bandung 40164, Indonesia; c.keiko.g@gmail.com; 2 Department of Clinical Pathology, Faculty of Medicine, Maranatha Christian University, Bandung 40164, Indonesia; 3 Department of Clinical Pathology, Faculty of Medicine, Padjajaran University—Hasan Sadikin General Hospital, Bandung 40161, Indonesia; agnes.indrati@unpad.ac.id

**Keywords:** human immunodeficiency virus, tuberculosis, co-infection, immune dysregulation, immune exhaustion, T cell senescence

## Abstract

Immune dysregulation is a hallmark of human immunodeficiency virus (HIV) infection, characterized by persistent immune activation and systemic inflammation that drive T cell exhaustion and senescence, contributing to disease progression and non-AIDS comorbidities, most notably tuberculosis (TB). With rising HIV prevalence, the incidence of HIV-TB co-infection continues to rise, highlighting the need to understand their immunopathological interplay. This narrative review aims to examine the association between immune dysregulation in HIV-TB co-infection, with a focus on cytokine profiles and immunological biomarkers. Relevant literature was retrieved from multiple databases, with evidence demonstrating differential expression of cytokines—IL-17A, IFN-γ, TNF, IL-10, IL-6, IL-4, and IL-2—and T cell activation markers, such as CD38 and HLA-DR on CD4^+^ T cells in latent and active TB among HIV-infected individuals. These immune mediators are consistently co-expressed at higher levels in active TB compared to latent TB, suggesting heightened immune activation of both innate and adaptive immune responses in HIV-TB co-infection. However, these findings are largely based on observational data, and the precise mechanism by which cytokine and T cell biomarker dysregulation contributes to HIV-TB pathogenesis remains incompletely understood, underscoring the need for larger, mechanistic studies to address these gaps in the pathogenic pathway.

## 1. Introduction

Human immunodeficiency virus (HIV) remains one of the most consequential infectious diseases due to its profound and lasting impact on the immune system [1]. Rather than simply depleting immune cells, HIV drives a complex cascade of dysregulation, marked by chronic activation, persistent inflammation, and progressive loss of immune balance, which together compromise host defense and increase susceptibility to opportunistic infections [2,3]. The global scale of this problem underscores its significance, with over 1 million new infections occurring each year and around 40 million people currently living with HIV (PLHIV) worldwide, yet only about three-quarters having access to antiretroviral therapy (ART) [4,5,6]. Accumulating data indicate that individuals with untreated or ineffective ART have as much as a tenfold higher likelihood of progressing to active TB compared to HIV-uninfected individuals, reflecting both an elevated propensity for latent TB reactivation and increased susceptibility to acquiring new Mtb infection [7,8,9].

The interaction between HIV and TB exemplifies a true “syndemic,” referring to two diseases acting synergistically to amplify morbidity and mortality [10,11]. Approximately 10% of PLHIV with latent TB will develop active TB disease each year. This co-infection contributes significantly to global TB mortality, with an estimated 1.25 million TB-related deaths annually, including about 161.000 deaths among PLHIV. TB remains the leading cause of death among PLHIV worldwide, demonstrating the disproportionate impact of HIV-associated TB disease [12].

Effective control of Mtb requires a well-orchestrated cellular immune response that balances pathogen clearance with limited tissue damage. Macrophages act as the primary host cells for Mtb [13,14]. Their anti-bacterial capacity depends heavily on activation by CD4^+^ T-helper (Th1) cells through cytokines such as IFN-γ and TNF-α, which promote phagolysosomal fusion and granuloma formation—the hallmark of TB immunity [15,16]. In parallel, cytotoxic CD8^+^ T cells contribute by recognizing Mtb antigen presented via class 1 molecules of the major histocompatibility complex (MHC) and release IFN-γ and TNF- α to enhance macrophage function [17,18]. However, CD8^+^ T cells also secrete regulatory cytokines such as IL-10 and TGF-β, which can paradoxically favor Mtb persistence by suppressing the protective immune response [17,19].

In contrast, HIV infection profoundly disrupts this finely tuned immune balance through CD4^+^ T cell depletion and functional impairment, compromising the production of IFN-γ and other key cytokines necessary for macrophage activation [20]. As a result, granuloma integrity is weakened, and Mtb containment becomes impaired [21]. This gives rise to central paradox: despite persistent immune activation and systemic inflammation, the host remains unable to provide antimicrobial protection. Hence, the immune system exists in a hyperactivated yet ineffective state—failing to eliminate Mtb while simultaneously promoting immunopathology. This paradoxical immune dysfunction underlies the increased susceptibility, rapid progression, and poor outcomes observed in HIV-TB co-infection [22,23,24,25].

Throughout this review, immune dysregulation is interpreted within commonly recognized stages of HIV and TB infection, including untreated HIV, ART-treated infection, and the period of immune reconstitution following ART initiation, as well as latent TB infection, active TB disease, and TB-associated immune reconstitution inflammatory syndrome (TB-IRIS). These stages are used as conceptual frameworks to aid interpretation, acknowledging heterogeneity in study populations across the literature.

Given its clinical significance, the immunological mechanisms driving this synergistic relationship remain incompletely understood. The paradox of heightened inflammation coupled with impaired antimicrobial protection—particularly in the context of disrupted T cell function, macrophage activation, and cytokine imbalance—remains an area of active investigation [26]. Understanding these disruptions is essential for identifying immunologic correlations of protection and improving therapeutic strategies. This narrative review aims to synthesize current evidence on how HIV alters the host immune response required for Mtb control, with particular attention to CD4^+^ T cell-mediated immunity, chronic immune activation, and altered cytokine signaling.

## 2. Foundational Immunopathogenesis of HIV and TB Infections

### 2.1. Immunopathogenesis of HIV Infection

The hallmark of acquired immunodeficiency syndrome (AIDS) pathogenesis is a progressive depletion of CD4^+^ T lymphocytes, accompanied by profound impairment of cellular immunity and increasing susceptibility to opportunistic infections [27]. Although AIDS typically manifests decades after initial infection, CD4^+^ T cell loss begins rapidly during acute infection and continues throughout the disease course [28]. Early hypotheses proposed that disease progression resulted from slow viral-mediated destruction; however, subsequent evidence demonstrated that HIV replication is continuous and dynamic, even during clinical latency [29].

The immunopathogenesis of HIV infection begins when the viral envelope glycoprotein gp120 binds to the host CD4 receptor, which serves as the primary site of viral attachment. This interaction induces a conformational rearrangement, exposing binding domains for specific chemokine receptors—mainly CCR5 and CXCR4 [30]. A major advance in understanding HIV pathogenesis was the discovery of the chemokine co-receptors—CCR5 and CXCR4—which mediate viral entry [31,32,33]. In early infection, CCR5-tropic (R5) viruses preferentially target and destroy memory CD4^+^ T cells in mucosal and lymphoid tissues. As infection progresses, CXCR4-tropic (X4) variants may emerge, expanding viral tropism to naïve and central-memory subsets and accelerating immune decline [34,35]. Yet, direct cytopathic effects alone cannot explain the magnitude of CD4^+^ T cell loss. Most CD4^+^ T cell deaths occur in bystander cells that are not productively infected [36]. In these cells, incomplete reverse transcription of HIV triggers accumulation of viral DNA intermediates, leading to caspase-1-dependent pyroptosis—a highly inflammatory form of programmed cell death that amplifies tissue damage and immune activation [35,37]. Productively infected cells undergo apoptosis, while uninfected activated T cells die through activation-induced cell death (AICD). CD8^+^ cytotoxic T lymphocytes further contribute to depletion by eliminating infected targets via perforin/granzyme and Fas–FasL pathways [38,39].

These mechanisms—direct viral killing, pyroptosis, apoptosis, and immune-mediated cytotoxicity—are compounded by chronic immune activation, microbial translocation from a compromised gut mucosa, and lymphoid tissue fibrosis [40,41]. Together, they erode the regenerative capacity of the CD4^+^ T cell compartment. Conceptually, CD4^+^ T cell depletion unfolds in three phases: (1) rapid destruction of CCR5^+^ effector-memory T cells during acute infection, (2) partial regeneration from central-memory precursors, and (3) eventual homeostatic collapse as central-memory pools are exhausted [27]. Thus, CD4^+^ depletion reflects a multifactorial process integrating direct viral cytopathology, bystander death, immune activation, and disrupted lymphoid architecture—all converging to drive immunodeficiency [27,42].

The chronic immune activation that contributes to CD4^+^ T cell depletion also underlies a gradual functional decline in surviving T cells, culminating in a state of exhaustion marked by impaired effector activity and proliferative capacity. T cell activation is a precisely orchestrated, multistep process governed by three sequential signals that ensure effective immune defense while maintaining self-tolerance: antigen recognition, co-stimulation, and termination [43]. Upon antigen recognition through the T cell receptor (TCR) interacting with MHC molecules on professional antigen-presenting cells (APCs), T cells receive a primary activation signal [44,45]. Full activation requires a second, co-stimulatory signal, most prominently the engagement of CD28 with its ligands CD80 or CD86, which drives proliferation, survival, and effector differentiation. Additional costimulatory receptors, such as CD27, OX40 (CD134), ICOS (CD278), CD40L (CD154), and CD226, further amplify and sustain T cell responses [43,46].

Following pathogen clearance, inhibitory signals are induced to restore immune homeostasis [47]. These are mediated by immune checkpoint receptors—including programmed cell death protein (PD-1), cytotoxic T-lymphocyte-associated antigen 4 (CTLA-4), lymphocyte activation gene 3 (LAG-3), and T cell immunoglobulin and mucin-domain-containing protein 3 (TIM-3)—which transiently limit T cell activity and prevent immunopathology [48,49,50]. During chronic infections such as HIV, persistent antigen exposure and inflammatory signaling drive sustained expression of these inhibitory pathways, leading to a progressive loss of T cell effector function known as T cell exhaustion [51,52]. This dysfunctional state is characterized by impaired proliferation, diminished cytokine production, and altered metabolic capacity. Among inhibitory receptors, PD-1 serves as a central mediator of exhaustion [53]. Upon engagement with its ligands, PD-L1 or PD-L2, PD-1 recruits the phosphatases SHP1 and SHP2 to the immunological synapse, resulting in dephosphorylation of key signaling intermediates—including CD3ζ, ZAP-70, PLC-γ1, and the CD28–PI3K–AKT pathway [54,55]. This attenuation disrupts downstream PKCθ, mTOR, and Ras/MAPK/ERK signaling, suppressing activation and promoting metabolic quiescence. The severity of exhaustion further correlates with co-expression of additional inhibitory receptors such as CTLA-4, LAG-3, TIM-3, TIGIT, 2B4 (CD244), and CD160 [43,56].

Functionally, exhausted CD8^+^ T cells undergo a hierarchical decline in activity: initial loss of IL-2 production and proliferative capacity is followed by reduced polyfunctionality and TNF-α secretion, culminating in severely diminished IFN-γ production and cytotoxic potential. Although initially defined in CD8^+^ T cells, exhaustion also affects CD4^+^ T cells in chronic HIV infection. Exhausted CD4^+^ T cells exhibit impaired production of IL-2, IFN-γ, and TNF-α, as well as reduced helper function, further exacerbating CD8^+^ T cell dysfunction and perpetuating immune dysregulation. Multiple factors contribute to the onset and maintenance of T cell exhaustion in HIV. Continuous viral replication sustains antigenic stimulation, while chronic immune activation and inflammation create a suppressive environment. Immunoregulatory cytokines such as IL-10—induced by persistent type I and II interferon signaling—further inhibit T cell proliferation and effector cytokine synthesis. Collectively, these factors establish a self-reinforcing loop of immune activation, checkpoint upregulation, and functional decline that impairs effective viral control [43].

The chronic antigenic stimulation and immune activation that drive T cell exhaustion in HIV also accelerate immunosenescence, a process of premature immune aging marked by irreversible cell-cycle arrest and loss of proliferative potential. Under physiological conditions, cellular senescence serves as a protective mechanism to prevent genomic instability; however, in chronic HIV infection, this process becomes pathologic [57]. CD4^+^ T cells are inherently long-lived and undergo repeated rounds of activation and proliferation, rendering them particularly susceptible to replicative stress and telomeric attrition. Notably, the progressive decline of T cell proliferation observed during aging occurs alongside gradual telomere shortening—a key hallmark of cellular senescence [58].

In chronic HIV infection, persistent immune activation and sustained antigenic stimulation impose continuous proliferative and oxidative stress on T lymphocytes, driving premature cellular senescence [59]. Under physiological conditions, telomeres shorten gradually by 50–100 base pairs per cell division, serving as a molecular indicator of replicative history and a safeguard against uncontrolled proliferation. In contrast, during chronic HIV infection, the gradual loss of telomeres accelerates up to nearly 250 base pairs per division, due to persistent inflammatory signaling and metabolic dysregulation [60]. When telomeres reach a critically short length, affected cells undergo irreversible growth arrest and acquire a senescent phenotype. This process is compounded by defective DNA repair mechanisms, which amplify telomeric instability and contribute to replicative exhaustion, diminished immune renewal capacity, and progressive CD4^+^ T cell depletion [57,61].

Viral proteins encoded by HIV further potentiate this senescent state [62]. The Tat protein induces NF-κB-mediated oxidative stress and mitochondrial dysfunction, while Nef disrupts autophagy pathways, impairing cellular quality control and accelerating telomere damage [63,64]. Through these mechanisms, HIV actively promotes cellular stress responses that compromise T cell viability and perpetuate chronic immune dysfunction. Senescence-associated changes are not confined to CD4^+^ T cells but are also evident in CD8^+^ T cell populations, which exhibit shortened telomeres, reduced telomerase activity, and increased expression of markers such as CD57 and KLRG1 [65,66,67]. Notably, decreased telomerase activity in CD8^+^ T cells correlates with elevated CD38 expression, reflecting heightened immune activation and serving as an indicator of disease progression. These findings highlight T cell senescence as both a hallmark and a pathogenic driver of immune deterioration in HIV infection, linking viral persistence to the accelerated aging of the adaptive immune system [57].

### 2.2. Immunopathogenesis of TB Infection

TB is currently recognized not only as an infectious disease, but also as an immune-mediated disorder in which disease outcome depends on the dynamic interaction between Mtb and the host immune system. Following exposure, approximately 90% of immunocompetent individuals with Mtb enter a latent infection state, while only about 10% of individuals progress to active TB infection (ATBI) [68]. The factors contributing to these outcomes reflect both bacterial virulence and host immune regulation. While Mtb lacks classical exotoxins, its pathogenicity lies in its ability to manipulate host immune responses through virulence factors that interfere with phagosome maturation, antigen presentation, and macrophage antimicrobial effector functions [69,70,71].

The immune response associated with the Mycobacterium tuberculosis infection (MTBI) begins with the innate immune system, which provides the first line of defense against the infection. Airway epithelial cells (AECs) and alveolar macrophages are among the earliest cells to encounter the pathogen [72]. Beyond its role as a physical barrier, AEC display several immunological function, recognizing Mtb through pattern recognition receptors (PRRs) such as Toll-like receptors (TLRs) and NOD-like receptors (NLRs). Their activation leads to the release of inflammatory mediators that recruit macrophages, neutrophils, dendritic cells (DCs), and natural killer (NK) cells to the site of infection [19].

Among these, macrophages play a central role as the first line of defense that functions to phagocytose cellular debris and pathogens in both fixed and free forms [73]. Upon recognition with Mtb components such as glycolipids and peptidoglycan via TLR2, TLR4, NOD2 and Dectin-1, the resident macrophages in the lungs engulf the bacilli and attempt to destroy the pathogen through phagolysosomal fusion, acid hydrolases, and the generation of reactive oxygen and nitrogen intermediates [71]. Beyond its role in phagocytoses, Mtb has evolved multiple mechanism resisting intracellular killing, including inhibition of phagosome–lysosome fusion and modulation of macrophage lipid metabolism, leading to the production of proinflammatory cytokines, such as TNF-α, IL-12, and IL-1β, further activating immune pathways [74,75,76].

As a key effector cell of innate immunity, natural killer (NK) cells are subsequently recruited and activated through cytokine- and receptor-mediated interactions. Through the secretion of IFN-γ and IL-22, NK cells potentiate macrophage antimicrobial activity, thereby restricting early mycobacterial replication [77]. In parallel, dendritic cells internalize mycobacteria and migrate to regional lymph nodes, where they present antigens to naive T cells. By upregulating CD80/CD86 and secreting IL-12, DCs drive the differentiation of CD4^+^ T cells toward the Th1 phenotype, bridging innate and adaptive immunity [78].

As infection progresses, these cellular interactions culminate in the formation of the tuberculous granuloma—a hallmark structure of TB immunopathogenesis [79]. The core of the granuloma consists of infected macrophages, epithelioid cells, and multinucleated giant cells surrounded by a mantle of CD4^+^ and CD8^+^ cells, B cells, neutrophils, and fibroblasts [80,81]. The functional role of granuloma, however, remains a subject of ongoing debate. While it serves as a protective barrier that confines Mtb and limits in dissemination, it may also facilitate bacterial persistence that contributes to disease progression and tissue damage. Within this microenvironment, factors such as hypoxia, nutrient limitation, and altered cytokine signaling promote immune evasion and metabolic adaptation of the pathogen that promote Mtb immune evasion. This balance between immune containment and bacterial persistence underlies the establishment of latent TB infection (LTBI)—a state of immune equilibrium in which viable Mtb persist without presenting clinical outcomes [19].

## 3. The Immunological Nexus: Dysregulation in HIV-TB Co-Infection

**Figure 1:**
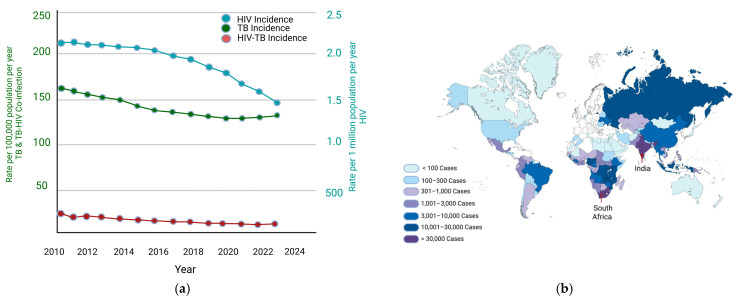
Global trends and geographic distribution of HIV-TB syndemic: (**a**) TB and HIV-TB co-infection are shown per 100,000 population per year, while HIV infection is presented per 1,000,000 population per year. (**b**) Global distribution of HIV-TB co-infection. Darker shades signify countries with the heaviest syndemic burden. Data adapted from World Health Organization estimates on TB Disease Burden, 2024 [82].

The immunological mechanisms discussed in this section primarily reflect HIV-TB co-infection in ART-naïve individuals or in those with incomplete viral suppression, where CD4^+^ T cell depletion and chronic immune activation are most pronounced. HIV-TB co-infection establishes a complex immunological interplay, in which the effects of HIV-induced CD4^+^ T cell depletion along with chronic immune activation converge with the immune mechanisms necessary for controlling TB infection. This intersection creates a syndemic, characterized by simultaneous immune dysregulation, systemic inflammation, and impaired pathogen-specific responses, ultimately increasing susceptibility to active TB [11]. The resulting syndemic reflects the dynamic interaction between viral and bacterial pathogens, shaping both disease progression and the host immune landscape (Figure 1).

### 3.1. Compromised Granuloma Function

CD4^+^ T cell depletion is one of the defining features of HIV infection [80,83]. CD4^+^ T cells serve as the central facilitators of both cellular and humoral immune responses and are maintained by tightly upregulated homeostatic mechanisms. HIV selectively targets these cells—the central coordinators of adaptive immunity—by binding to the CD4 receptors, entering the cell and replicating within it [29,84]. The resulting viral replication not only destroys infected cells but also induces bystander death of surrounding uninfected T cells through inflammatory and immune-mediated pathways. As this relentless cycle of destruction outpaces cellular renewal, the immune system gradually loses its capacity to maintain homeostasis, resulting in CD4^+^ T cell depletion [29,41,42].

Paradoxically, the formation and stability of granulomas—defining features of Mtb infection—depend critically on CD4^+^ T cells [85,86,87]. Granulomas are organized aggregates of macrophages and other myeloid cells—such as DCs, neutrophils, eosinophils, multinucleated giant cells, and mast cells—encircled by lymphocytes, including T cells, B cells, NK cells, and innate lymphoid cells [88,89,90,91]. A positive association has been reported between the abundance of CD4^+^ T cells in granulomatous tissue and the structural integrity of granuloma, indicating that higher CD4^+^ T cell presence promotes well-formed granulomas and effective bacterial containment, intertwined with the host’s immune competence [92,93].

In the setting of HIV-TB co-infection, the dual role of CD4^+^ T cells—as both the primary targets of HIV and the key mediators of granulomas in TB immunity—becomes critically evident. HIV-driven CD4^+^ T cell depletion, coupled with macrophage and dendritic cell dysfunction, disrupts granuloma architecture and compromise bacterial control [94,95,96,97]. HIV-TB co-infection has been shown to reduce the proportion of CD4^+^, CD3^+^, and CD8^+^ T cells within granulomatous tissues, resulting in fewer lymphocytes available to sustain the granulomatous response [98,99,100]. Moreover, Mtb-specific pulmonary CD4^+^ T cells expressing IFN-γ and TNF-α cytokines essential for macrophage activation and bacterial containment are markedly depleted in HIV-infected individuals [96]. The inflammatory environment within granulomas enhances HIV replication. Granulomas contain multiple HIV-susceptible cell types, expressive CCR5 and CXCR4, facilitating local viral propagation [101,102]. This localized viral expansion accelerates CD4^+^ T cell loss within the granuloma, disturbing the delicate balance between pro- and anti-inflammatory cytokines required for bacterial control. In addition, early CD4^+^ T cell depletion irreversibly disrupts granulomatous T cell composition, and even after partial peripheral immune reconstitution, granulomas fail to recover their structural integrity, leading to increased bacterial growth, sustained inflammation, and continued recruitment of target cells that support HIV replication, creating a vicious cycle that exacerbate both HIV-TB infections [103,104].

**Figure 2:**
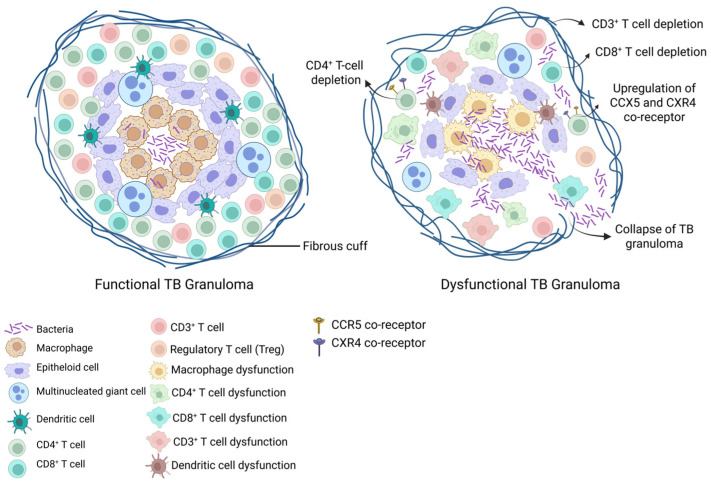
Functional vs. dysfunctional TB granuloma. The functional granuloma shows a well-organized structure with activated macrophages, epithelioid cells, multinucleated giant cells, dendritic cells, and abundant CD4^+^ and CD8^+^ T cells that work together to contain Mycobacterium tuberculosis. In contrast, the dysfunctional granuloma exhibits marked CD4^+^ and CD3^+^ T cell depletion, macrophage dysfunction, and disrupted immune cell layering, allowing collapse of TB granuloma. Original schematic illustration created for this study.

In HIV-infected individuals exposed to Mtb, impaired phagocytosis by macrophages and dendritic cells hinders bacterial clearance, while reduced antigen presentation delays T cell activation and recruitment to infection sites. Consequently, granulomas formed under these conditions are poorly organized and inefficient at containing bacteria (Figure 2). HIV-induced macrophage dysfunction further limits bacterial killing capacity, amplifying bacterial replication and viral spread. The breakdown of granuloma structure prevents the establishment of latent TB infection and instead promotes dissemination and progression to ATBI [97,105,106]. This breakdown in granuloma integrity reflects the central paradox of HIV-TB co-infection: a state of profound cytokine dysregulation in which the immune system is intensely hyperactivated yet unable to control infection effectively.

### 3.2. Dysregulation of Cytokine Networks

HIV infection leads to a profound deterioration of immune function, notably through the depletion of CD4^+^ T cells and impairment of macrophage activity, thereby comprising the host’s capacity to contain Mtb growth. Conversely, Mtb infection adversely impacts the immune system in individuals with HIV, accelerating the progression from HIV infection to AIDS. This process is mediated by pro- and anti-inflammatory cytokines that trigger signaling cascades in T cells and monocytic cells, ultimately enhancing HIV transcription through the activation of the of transcription factors NF-kB (Nuclear factor-kB) and NFAT (Nuclear factor of activated T cells) [107,108]. Under physiological conditions, an effective immune response depends on a delicate equilibrium between pro-inflammatory and regulatory (anti-inflammatory) cytokines. This balance ensures pathogen clearance while preventing excessive inflammation [109]. However, in the case of HIV-TB co-infection, this balance is profoundly disturbed, leading to an immune response that favors disease progression rather than protection [26].

In the context of HIV and TB infection, both pathogens exhibit disrupt cytokine signaling, yet their coexistence results in a deeper and more persistent state of immune dysregulation [110,111]. During Mtb infection, T cell differentiation within granulomatous foci leads to increased expression of the CCR5 and CXC4 co-receptors on the surface of CD4^+^ T cells, enhancing their susceptibility to HIV infection, as these co-receptors mediate viral entry into host cells [112]. Notably, Mtb-specific CD4^+^ T cells are particularly prone to HIV-mediated depletion compared with naive cells, resulting in a localized loss of antigen-specific T cells at the site of infection. This heightened susceptibility is largely attributed to the upregulation of CCR5 on CD4^+^ T cells and other immune cell subsets following Mtb infection [107].

Physiologically, CCR5 mediates immune cell recruitment to infection sites, promoting granuloma organization and effective antimycobacterial activity. However, as CCR5 serves as a principal co-receptor for HIV, its upregulation inadvertently promotes viral access and accelerates CD4^+^ T cell depletion [113]. Conversely, Mtb infection induces sustained cytokine release to promote granuloma maintenance and bacterial containment, often accompanied by increased CD4^+^ T cell recruitment. When both pathogens coexist, their immunopathological mechanisms converge synergistically—Mtb-driven CCR5 upregulation amplifies HIV infectivity, while HIV-mediated CD4^+^ depletion weakens Mtb control. The outcome is a self-perpetuating cycle of immune activation and cytokine imbalance that undermines bacterial containment and simultaneously facilitates viral replication and dissemination [11].

A key feature observed in HIV-TB co-infection is the disruption of the Th1/Th2 cytokine balance [114]. HIV-TB co-infected individuals exhibit markedly reduced levels of IFN-γ, TNF-α, and IL-2 compared to patients with either infection alone [115]. These cytokines are central to macrophage activation, T cell proliferation, and granuloma integrity. IFN-γ promotes classical macrophage activation, enhances phagolysosomal fusion, and upregulates MHC-II expression, thereby improving antigen presentation and cytotoxic T cell responses [116,117]. Genetic defects in IFN-γ signaling or its receptor results in impaired granuloma formation and heightened susceptibility to MTb infections, underscoring its pivotal role. A decline in IFN-γ during HIV-TB co-infection therefore translates into defective macrophage function and diminished containment of Mtb within granulomas [118].

TNF-α, produced by activated macrophages and T cells is another indispensable cytokine in TB immunity [119]. It facilitates granuloma formation by promoting cell recruitment and enhancing macrophage bactericidal activity [120]. At the same time, TNF-α plays a paradoxical role in HIV infection. While serving protection against Mtb, it activates the NF-κB transcription pathway, enhancing HIV replication within macrophages and T cells [121]. Nevertheless, HIV-TB co-infection has been associated with reduced TNF-α production, leading to weaker granulomatous responses and accelerated bacterial proliferation [115].

Similarly, IL-2 supports T cell clonal expansion, NK cell activation, and the functional maturation of cytotoxic lymphocytes, contributing to the containment of both viral and bacterial infections responses [122]. Yet, IL-2 can also stimulate HIV replication in activated T cells, creating a complex feedback loop that further distorts immune regulation. In HIV–TB co-infection, decreased IL-2 levels reduce effector cell proliferation and antigen-specific responses, while excessive viral replication continues to fuel immune exhaustion [123,124].

In contrast, regulatory cytokines such as IL-4 and IL-10 are often upregulated to counterbalance excessive inflammation [43,125,126]. However, in HIV-TB co-infection, this compensatory mechanism becomes maladaptive. IL-4, produced by activated CD4^+^ T cells, basophils, and mast cells, promotes Th2 polarization and suppresses macrophage-mediated killing [115,127]. Elevated IL-4 expression has been linked to enhanced susceptibility to Mtb infection, as it downregulates the Th1 response required for bacterial clearance. Although certain IL-4 gene polymorphisms have been associated with slower HIV progression through reduced viral replication, excessive IL-4 activity compromises antimycobacterial immunity by shifting the cytokine landscape toward a Th-2 dominant profile [128].

IL-10, a potent anti-inflammatory cytokine secreted by monocytes, macrophages, dendritic cells, and regulatory T cells, further exacerbates this dysfunction. It inhibits the synthesis of pro-inflammatory mediators and downregulates MHC-II expression on antigen-presenting cells, effectively dampening T cell activation [129]. High IL-10 concentrations observed in HIV–TB co-infected individuals suppress macrophage activation and impair bacterial killing, aggravating disease progression. Moreover, reduced IL-10 clearance and persistent immune activation perpetuate chronic immune dysfunction, reinforcing the cycle of inadequate pathogen control and sustained inflammation [130].

While key Th1 cytokines are suppressed, several other inflammatory mediators—such as IL-8, IL-12, and IL-18—are often elevated during HIV–TB co-infection, reflecting a compensatory yet pathological hyperactivation of the immune system [108,131]. IL-8 acts as a chemokine that recruits T cells and neutrophils to granulomatous sites, but its overexpression in HIV infection may protect infected cells from apoptosis, facilitating viral persistence [132,133]. IL-12, primarily produced by dendritic cells and macrophages, is essential for the differentiation of naive CD4^+^ T cells into Th1 effectors and for the induction of IFN-γ production [134]. However, in the setting of HIV–TB co-infection, this pathway becomes dysregulated—elevated systemic IL-12 coexists with reduced local IFN-γ activity, suggesting functional disconnection between cytokine signaling and cellular responses [108,135]. Similarly, IL-18, a pro-inflammatory cytokine that enhances IFN-γ production and CD8^+^ T cell cytotoxicity, is frequently upregulated in HIV–TB patients. Paradoxically, excessive IL-18 can induce HIV replication, promote immune hyperactivation, and contribute to CD4^+^ T cell depletion, accelerating immunopathogenesis [108,136].

This complex cytokine disequilibrium—characterized by suppressed Th1 cytokines (IFN-γ, TNF-α, IL-2) alongside aberrant increases in IL-8, IL-12, and IL-18—undermines granuloma stability and alters the immune regulation toward chronic, ineffective inflammation [108]. The concurrent reduction in Th17 and gamma delta (γδ) T cell populations, both important sources of IL-17 and other pro-inflammatory mediators, further diminishes mucosal immunity and bacterial control [137]. IL-17 deficiency impairs neutrophil recruitment and epithelial defense, allowing greater Mtb dissemination and exacerbating tissue pathology [138]. Consequently, the immune response in HIV–TB co-infection transitions from a protective, localized reaction to a disseminated, pathological inflammation incapable of achieving bacterial containment or viral suppression.

### 3.3. T Cell Phenotypes as Markers of Disease

Beyond the disruption of soluble cytokine networks, the paradox of immune hyperactivation alongside functional ineffectiveness is vividly manifested in the altered phenotypes of T cells. Alterations in T cell phenotypes have been found to disrupt the delicate balance between pro- and anti-inflammatory responses. Within granulomatous structures that contain Mtb in latency, T cell populations serve as key immunological components. In parallel, HIV-induced dysregulation of these T cells can compromise granulosa integrity, leading to its disorganization and subsequent release of the pathogen into the lungs, resulting in increased proliferation in the absence of an effective immune response [139]. Despite their essential role in host immunity, shifts in T cell populations during HIV-TB co-infection are considered major regulatory drivers of the disease’s immunopathogenesis [140].

In the context of HIV co-infection, however, this protective axis is severely disrupted. Activated CD4^+^ T cells upregulate CCR5 and CXCR4, enhancing their susceptibility to viral entry [35]. Consequently, Mtb-specific CD4^+^ T cells are preferentially depleted, compromising granuloma stability and local immune control, while the loss of IL-2-producing cells accelerates viral replication, creating a self-reinforcing cycle of immune depletion [15]. While compensatory proliferation initially replenishes CD4^+^ cell populations, persistent antigenic stimulation and chronic inflammation gradually drive these cells toward functional exhaustion—exhibiting impaired proliferation, reduced secretion of IL-2 and IFN-γ, and skewing toward terminal effector- or regulatory-like phenotypes [8].

Persistent engagement of inhibitory receptors—including PD-1, TIM-3, and LAG-3—not only disrupts TCR signaling but also reprograms downstream metabolic pathways [141]. Specifically, PD-1 upregulation inhibits PI3K/Akt signaling, reduces glucose uptake, and compromises mitochondrial respiration, collectively increasing susceptibility to apoptosis [142]. Additional changes in the surface receptors further reinforce the exhausted state. Downregulation of CD127 limits survival signaling and memory maintenance, while sustained CD38 expression signals chronic activation and systemic inflammation. Meanwhile, transcription factors such as BATF, IRF4, and TOX reshape the chromatin landscape, driving CD4^+^ T cells toward a hyporesponsive, exhausted state that persist even after antigen clearance [143]. In the co-infection, this exhaustion further undermines Th1 responses, compromises macrophage activation, and diminishes mucosal barrier integrity, creating a permissive environment for both bacterial persistence and viral replication.

While CD4^+^ T cells play a role in early activation and are susceptible to exhaustion, studies on T cell exhaustion have predominantly centered on CD8^+^ T cells [104]. CD8^+^ T cells, as the cytotoxic arm of adaptive immunity, complement helper functions by eliminating Mtb-infected macrophages and virally infected cells. Activation of CD8^+^ T cells is initiated through MHC class 1-restricted antigen presentation by dendritic cells and infected macrophages, driving differentiation into effector cytotoxic T lymphocytes (CTLs) capable of secreting perforin, granzyme B, and IFN-γ [144]. Under chronic antigen exposure, persistent Mtb or HIV infection drives CD8^+^ T cells toward functional exhaustion. Initially, these cells proliferate and secrete cytokines efficiently; however, sustained stimulation imposes a continuous demand, promoting terminal differentiation at the expense of naive and central memory pools [17,145,146]. Mtb-specific CD8^+^ T cells exhibit bioenergetic insufficiency, with impaired oxidative phosphorylation and limited glycolytic flexibility, reducing cytotoxic capacity [147].

In parallel, continuous HIV exposure similarly reduces proliferative capacity, IL-2 and IFN-γ production, and β-catenin-dependent TCF transcriptional programs critical for maintenance of stem-like memory subsets, ultimately restricting long-term immune surveillance [43,148,149]. Similarly to CD4^+^ T cells, exhaustion is marked by upregulation of inhibitory receptors, including PD-1, TIM-3, LAG-3, and CTLA-4, which collectively suppress TCR signaling, cytokine secretion, and recall responses [150]. Phenotypically, exhausted CD8^+^ T cells downregulate costimulatory molecules (CD28, CD27) while expressing terminal differentiation and senescence markers such as CD57, alongside CD127 loss, indicating compromised survival and proliferative potential. The CD57^+^CD127^−^ subset in co-infected individuals sustains inflammatory cytokine production (TNF-α, IL-6) despite functional impairment, fostering a microenvironment permissive for pathogen persistence [108].

**Figure 3:**
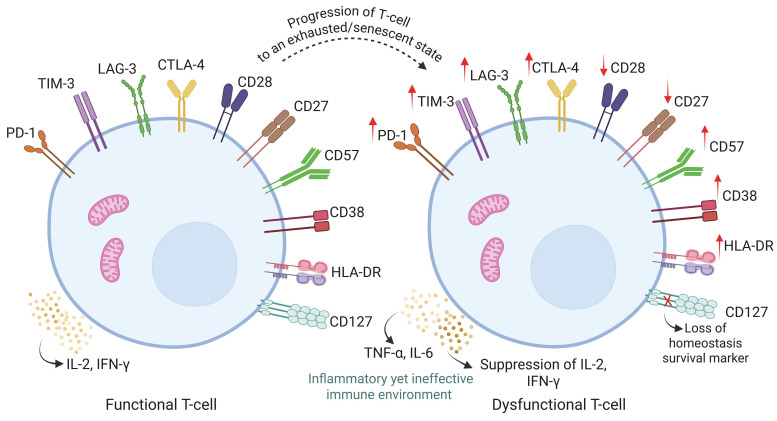
Transition from functional to dysfunctional T cells. The functional T cells express balanced co-stimulatory (CD28 and CD27) and inhibitory receptors (PD-1, TIM-3, LAG-3, and CTLA-4), producing IL-2 and IFN-γ to sustain effective immunity. The shift to dysfunctional T cells revealed an upregulation of inhibitory receptors (PD-1, TIM-3, LAG-3, CTLA-4), activation markers (CD38, HLA-DR), and senescence markers (CD57). This drives progression toward an exhausted/senescent state, resulting in an inflammatory yet ineffective immune environment. Arrows (↑/↓) indicate increased/decreased expression of these markers during the transition. Original schematic illustration created for this study.

The unconventional T cell, encompassing a variety of cell subsets, including mucosal-associated invariant T (MAIT) cells, gamma delta (γδ) T cells, and invariant natural killer T (iNKT) cells, is linked to providing innate-like rapid responses to microbial antigens [151,152]. MAIT cells, recognizing riboflavin-derived metabolites via MR1, are early responders during Mtb infection, producing IFN-γ, TNF-α, IL-17, and IL-22 [153]. Conversely, MAIT cells are numerically depleted and functionally impaired in HIV-TB co-infection. Persistent antigen exposure induces PD-1 upregulation and dysregulated chemokine receptor expression, limiting trafficking and predisposing MAIT cells to exhaustion [154,155]. γδ T cells, particularly the Vδ2 subset, respond independently of MHC to phosphoantigens with cytotoxic and cytokine-mediated antimicrobial activity [156]. Chronic co-infection depletes circulating Vδ2 cells, expands tissue-resident Vδ1 populations, and induces aberrant CD4^+^CD8^+^ co-expression alongside reduced effector function. Elevated PD-1 and diminished CD127 expression denote progressive exhaustion, while sustained HLA-DR and CD38 reflect ongoing activation. Similarly, iNKT cells, which recognize glycolipid antigens via CD1d, are depleted among CD4^+^CD8^−^ subsets and show expansion of CD4^−^CD8^+^ and double-negative populations. Despite reduced numbers, these cells maintain CD107a expression and secret proinflammatory mediators, amplifying CD4^+^ T cell activation and neutrophil recruitment, thereby contributing to tissue immunopathology [157] (Figure 3).

## 4. Immunological Spectrum: Active vs. Latent TB in HIV Co-Infection

### 4.1. Immunological Hallmark of Active TB Infection (ATBI)

The immunological distinctions between latent and active TB in PLHIV are shaped not only by mycobacterial burden, but also by HIV disease stage and ART status, which modulate the magnitude and quality of host immune response. The gradual depletion of naive and memory CD4^+^ T cells are the ultimate hallmark of HIV infection [36]. HIV infection alters the T helper (Th) cell equilibrium by suppressing Th1-mediated responses and enhancing Th2 activity—a phenomenon described as the Th1/Th2 shift. This imbalance arises from the preferential replication of HIV within Th2-like cells, which favor viral persistence more effectively than Th1 cells. The viral envelope glycoproteins gp120 and gp160 play critical roles in this process. By binding to the CD4 receptor, gp120 triggers Th2-associated IL-4 production, which inhibits Th1 differentiation and promotes IgE synthesis [158]. Furthermore, gp120 contributes to oxidative stress through the upregulation of cytochrome P450 2E1 (CYP2E1), enhancing the generation of reactive oxygen species (ROS). The resulting oxidative damage, including telomeric DNA injury, promotes tissue injury and apoptosis [159]. Concurrently, HIV-induced cell death occurs through both caspase-3-dependent apoptosis in permissive CD4^+^ T cells and caspase-1-mediated pyroptosis in non-permissive cells, amplifying local inflammatory responses [160].

The reduction in CD4^+^ T cells significantly weakens immune defense, predisposing individuals to opportunistic infections such as Mtb. Inhaled as infected droplets, Mtb primarily infects alveolar macrophages, initiating localized inflammatory signaling that recruits macrophages, neutrophils, and other immune cells to form granuloma—a characteristic of latent TB infection (LTBI) [14,161]. Within granulomas, the coordinated activity of CD4^+^ and CD8^+^ T cells, together with cytokines, maintains a dynamic balance between bacterial containment and immune-mediated tissue injury [162].

In the case of HIV-TB co-infection, as HIV progresses, this equilibrium progressively shifts. As HIV replication advances, peripheral CD4^+^ T cell (pCD4) depletion disrupts granuloma organization, resulting in coexistence of well-structured and poorly formed granulomas [159,163]. The concurrent reduction in CD8^+^ T cell cytotoxicity and IFN-γ production weakens macrophage activation, allowing Mtb replication and increased bacterial burden [126]. In parallel, the cytokine general environment becomes increasingly dysregulated; notably, TNF-α and IL-6 are upregulated, while the regulatory cytokine IL-10 is downregulated, fostering a hyperinflammatory environment that supports both HIV and Mtb persistence. Chronic antigenic stimulation and sustained inflammation lead to upregulation of immune checkpoint receptors—including PD-1, CTLA-4, LAG-3, and TIM-3—on T cells, further driving functional exhaustion [164,165]. Collectively, these processes delineate the immunological spectrum of TB in the context of HIV co-infection, driving the progression from latent to active disease. The continuous deterioration of immune regulation permits increasing Mtb replication, enhanced cytokine dysregulation, and systemic hyperinflammation—culminating in active TB disease, driven by this immune dysfunction [165].

### 4.2. Immunological Hallmark of Latent TB Infection (LTBI)

Latent tuberculosis infection (LTBI)—a state of persistent immune response to stimulation by Mtb without evidence of clinical manifestations—has a distinct immune–inflammatory profile, characterized by a functionally competent, controlled Th1 response and Mtb-specific T cells [164,166]. This immune landscape reflects a delicate balance between proinflammatory and regulatory mechanisms that enable long-term bacterial containment while minimizing immunopathology.

Individuals with LTBI exhibit a distinct immune–inflammatory profile featuring functionally active Mtb-specific memory CD4^+^ and CD8^+^ T cells with maintained cytolytic potential and controlled activation status [164,166]. Rather than global immune activation, LTBI demonstrates selective and efficient T cell responses, ensuring pathogen control without collateral tissue damage. This containment is supported by an intricate regulatory network mediated largely by regulatory T cells (Tregs), which preserve immune homeostasis and prevent excessive inflammation [167]. In the case of LTBI, both CD4^+^ and CD8^+^ Tregs have been recognized to contribute to this regulation. CD4^+^ Tregs, defined by CD4^+^CD25^+^FOXP3^+^ expression, employ both contact-dependent and -independent mechanisms that suppress effector T cell activity. These mechanisms include modulation of APCs through CTLA-4 engagement, suppressing multiple cell types through secretion of anti-inflammatory cytokines such as IL-10, TGF-β, and IL-35 and depletion of IL-2, which limits effector T cell proliferation [168,169]. In parallel, CD8^+^ Tregs, a heterogenous population of CD8^+^ lymphocyte-expressing regulatory markers—CD122, CD25, CD103, GITR, CTLA-4, and PD-1—exert inhibitory effects through similar pathways, releasing IL-10 and TGF-β, as well as inhibitory cell-to-cell interaction through CTLA-4 and PD-1 signaling pathways, and have the immunosuppressive capacity to engage in cell contact-dependent and -independent mechanisms to suppress immune response [169,170,171] (Table 1).

Furthermore, LTBI displays differential expressions of immune checkpoint and regulatory genes that reinforce this balanced state. Upregulation of CD44, TIGIT, and LAG3, alongside reduced expression of IDO2, ADORA2A, LAIR1, and TNFSF14, indicates a regulatory profile that favors effective pathogen control while limiting excessive inflammation. CD44 has been implicated in shaping immune responses through its influence on macrophage polarization and the recruitment of immune cells, facilitating granuloma stability, whereas LAG3 and TIGIT serve as inhibitory checkpoints, preventing overactivation and terminal T cell exhaustion [172]. These regulatory adjustments allow LTBI to sustain Th1-mediated bacterial control with limited systemic inflammation.

Cytokine modulation also contributes significantly to this equilibrium. TNF-α influences granuloma integrity and limits bacterial dissemination, while IL-12 regulates IFN-γ production and the cytotoxic activity of T cells [173]. Conversely, anti-inflammatory cytokines such as TGF-β1 and IL-10 have been shown to regulate lymphoid and myeloid derived cells, susceptible to mycobacterial persistence in TB infection [135,174].

Progression from LTBI to ATBI reflects a collapse of this immune balance. As LTBI transitions to ATBI, the systemic inflammation intensifies, reflected by elevated levels of C-reactive protein (CRP), IL-6, and TNF-α and heightened expression of CD4^+^ and CD8^+^ T cells expressing HLA-DR and CD38 [24,174]. Chronic antigen exposure combined with sustained inflammatory signaling drives T cell dysfunction and exhaustion, marked by increased PD-1, TIM-3, and LAG-3 expression and reduced IFN-γ/IL-2 co-expression [26,175,176]. Moreover, Th1 and TH17 cytokines (IFN-γ, TNF and IL-2, and IL-17a) are markedly elevated, while Th2 cytokines (IL-4, IL-10, and IL-6) contribute to the counterproductive anti-inflammatory environment, particularly the setting of co-infection with HIV [26,175]. This cytokine disequilibrium disrupts granuloma structure and facilitates bacterial reaction. Moreover, increased IL-6 activity enhances monocyte expansion and bacterial growth, linking systemic inflammation directly to disease activity [177].

**Figure 4:**
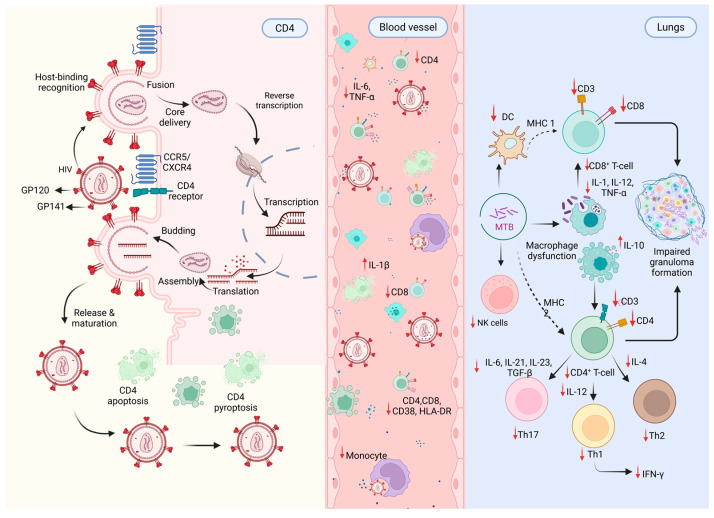
HIV infection promotes susceptibility to Mycobacterium tuberculosis through multilevel immune disruption. HIV-driven CD4+ T cell depletion and chronic immune activation impair both innate and adaptive immune responses, accompanied by increased pro-inflammatory cytokine production. In the lung microenvironment, dysfunctional macrophage and dendritic activity, impaired antigen presentation, and altered T helper cell differentiation compromise effective granuloma formation. These immune abnormalities facilitate Mtb persistence and disease progression during HIV-TB co-infection. Arrows indicate the direction of cellular interactions and trafficking; upward (↑) and downward (↓) arrows denote relative upregulation or downregulation of immune pathways and mediators. Original schematic illustration created for this study.

The contrasting immunological landscapes of LTBI and ATBI highlight potential biomarkers for disease classification. Elevated CRP, soluble urokinase plasminogen activator receptor (suPAR), and IL-6 levels correlate strongly with ATBI, which strongly reflects systemic inflammation and immune activation [177,178,179]. Similarly, sCD14 and increased T cell activation markers such as CD38 and HLA-DR are associated with active disease, particularly in HIV-co infection, where they parallel decreased CD4^+^ cell counts (Figure 4) (Table 1) [24]. Collectively, these cytokines and immune mediators contribute to the transition from LTBI to ATBI, amplifying inflammation and driving disease progression.

## 5. Clinical Implications and Future Directions

The extensive immune dysregulation outlined above extends far beyond mechanistic insight and translates into pressing clinical challenges. This is reflected in the inconsistent performance of conventional diagnostic approaches and the emergence of severe inflammatory complications in HIV-TB co-infection.

### 5.1. Impact on Diagnostics and Prognosis

The distinct immunoinflammatory signatures observed in LTBI and ATBI underscore critical challenges in current diagnostic strategies, particularly in PLHIV. Conventional tools such as the tuberculin skin test (TST) and interferon -γ release assays (IGRAs), although routinely employed for latent TB screening, have limited diagnostic accuracy in ATBI [180]. Their reliance on a functional host immune response becomes their primary weakness in HIV-TB co-infection. The very T cell depletion and exhaustion (e.g., loss of IFN-γ-producing cells) that defines the co-infection pathogenesis causes these tests to frequently yield false-negative or indeterminate results, failing to distinguish active from latent infection [181,182].

Similarly, bacteriological confirmation remains suboptimal. Low bacterial loads in sputum (paucibacillary disease) are common in immunocompromised hosts, reducing the sensitivity of smear microscopy and even molecular assays [182,183]. This diagnostic gap highlights the pressing need for biomarkers that capture the underlying pathological activity rather than merely indicating immune sensitization, a direction supported by the immune mediators outlined in the review. Moving beyond single-analyte assays, integrated biomarker signatures may offer substantially greater diagnostic and prognostic precision. Elevated systemic inflammatory markers such as IL-6, CRP, and suPAR are strong indicators of the pathological and ineffective inflammation that defines ATBI, in contrast to the more regulated immune state of latent infection. Similarly, quantification of T cell activation (CD38^+^ and HLA-DR^+^) and exhaustion (PD-1^+^) markers may generate a prognostic signature of immune dysfunction that closely aligns with disease progression [184,185,186]. The multi-dimensional nature of immune disruption in HIV-TB co-infection supports the use of a multi-biomarker approach that is likely to offer superior diagnostic performance compared to existing single-analyte assays, which frequently lack sensitivity or yield absent signals in immunocompromised individuals.

### 5.2. Therapeutic Considerations: TB-IRIS and Host-Directed Therapies

Following ART initiation, understanding the immune paradox of HIV-TB co-infection also reshapes the therapeutic framework, revealing significant challenges alongside opportunities for intervention. One of the most critical manifestations of this dysregulated immunity is TB-associated immune reconstitution inflammatory syndrome (TB-IRIS) [187]. This paradoxical deterioration following ART initiation reflects the abrupt reactivation of immune pathways—including TNF-α, IL-6, and IL-18—that were previously suppressed or functionally chaotic [188]. Elevated pre-ART inflammatory signatures and heightened T cell activation carry the greatest risk, illustrating that TB-IRIS constitutes an amplified expression of the very immunopathology described throughout this review [189].

**Figure 5:**
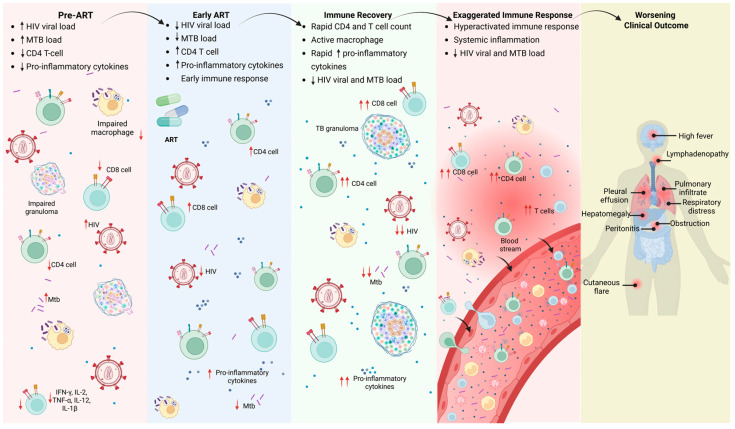
Immunological changes across stages of HIV-TB co-infection, from untreated HIV infection (pre-ART) to early ART initiation and subsequent immune recovery. In the absence of ART, high HIV viral load drives CD4^+^ T cell depletion, impairs macrophage function, and disrupts granuloma containment of Mycobacterium tuberculosis. ART initiation promotes rapid immune restoration, characterized by CD4^+^ T cell expansion and heightened pro-inflammatory cytokine responses. In a subset of susceptible individuals, this abrupt immune reconstitution triggers exaggerated inflammation, resulting in tissue damage and clinical manifestations of TB-IRIS. Upward arrows (↑) denote increased immune activation, and downward arrows (↓) denote decreased activation, while double upward arrows (↑↑) and double downward arrows (↓↓) indicate a more pronounced increase and decrease, respectively.

This evolving understanding underscores the potential role of host-directed therapies (HDTs) as an important adjunct to conventional antimicrobial treatment. HDTs shift the therapeutic focus from solely targeting MTB to correcting the underlying immune dysregulation that characterizes HIV-TB co-infection [190]. These approaches generally fall into two complementary categories: attenuating pathological inflammation, either through broad-acting anti-inflammatory agents or through selective cytokine inhibitors, and reinstating effective, pathogen-specific immunity, including the use of immune-modulating strategies such as checkpoint inhibitors [191,192,193]. Taken together, these insights suggest that future HIV-TB management will require a coordinated, multi-layered strategy: antibiotics to eliminate the bacillus, ART to restore CD4^+^ T cell function, and HDTs to fine-tune the immune response by promoting localized Th1-mediated protection while suppressing the excessive systemic inflammation that contributes to disease severity and TB-IRIS (Figure 5).

Notably, the dissociation between viral control, CD4+ T cell recovery, and effective antimycobacterial immunity is not exclusive to TB-IRIS, but is also observed in a distinct population of PLHIV known as elite controllers.

### 5.3. Insights from Elite Controllers: Implication in HIV-TB Immunopathogenesis

Elite controllers (ECs) represent immunological outliers that provide a unique opportunity to interrogate HIV-associated immune dysregulation beyond conventional disease trajectories. An intriguing perspective emerges from the study of ECs, a rare subset of PLHIV who maintain durable viral suppression in the absence of ART and preserve near-normal circulating CD4^+^ T cell counts [194]. Despite this favorable immunovirological profile, emerging evidence suggests that ECs remain susceptible to tuberculosis at rates comparable to non-controllers, highlighting that preservation of peripheral CD4^+^ T cell numbers alone is insufficient to confer protection against MTBI [195].

Although data on HIV-TB co-infection in ECs remain limited, emerging evidence indicates that ECs exhibit profound perturbations in monocyte homeostasis and phenotype despite effective viral control—including altered monocyte subset proportion and functional marker expression [196,197]. Given the central role of monocytes and macrophages in Mtb uptake, granuloma formation, and cytokine regulation, such qualitative innate immune perturbation could plausiby contribute to impaired antimycobacterial responses in ECs.

From a translational perspective, ECs represent a valuable natural model to disentangle mechanisms of HIV-associated immune dysregulation independent of profound CD4^+^ T cell depletion. Comparative studies examining granuloma architecture, cytokine networks, and tissue-resident immune responses in ECs versus ART-treated and untreated individuals may offer critical insights into immune correlates of protection and inform host-directed therapeutic strategies in HIV-TB co-infection [198].

## 6. Conclusions

HIV-TB co-infection remains a formidable syndemic, defined by a central immunological paradox in which chronic systemic inflammation coexists with profound functional immune exhaustion. As synthesized in this review, this paradox disrupts the very mechanisms required for pathogen control—undermining granuloma stability, distorting cytokine networks, and driving sustained T cell activation, dysfunction, and exhaustion. A clear understanding of these processes establishes the foundation for meaningful clinical advances. Such mechanistic insight enables the refinement of diagnostic strategies, supports the development of robust prognostic biomarker profiles, and guides the design of HDTs aimed at correcting the underlying immune dysregulation. A deeper understanding of immunopathological processes—rather than reliance on pathogen-centric strategies alone—will be fundamental in improving clinical outcomes in this deadly co-infection.

## Figures and Tables

**Table 1:** Immunological feature in active and latent TB in HIV-TB co-infection.

Features	ATBI	LTBI
Systemic inflammation	↑ IL-6, TNF-α, CRP, suPAR	Minimal systemic inflammation
T cell activation	↑ CD38^+^, HLA-DR^+^ in CD4 and CD8 T cell	Mild T cell activation
T cell exhaustion	↑ PD-1, TIM-3, LAG-3	Lower exhaustion marker
Cytokine profile	IL-2, IL-5, IL-6, IP-10, IL-13, IFN-γ, and TNF-α	Tightly regulated cytokine profile

ATBI (active TB infection); LTBI (latent TB infection). Upward arrows (↑) indicate increased expression of immune markers.

## Data Availability

The data are contained within the article.

## Abbreviations

The following abbreviations are used in this manuscript:

HIV	Human immunodeficiency virus
AIDS	Acquired immunodeficiency syndrome
TB	Tuberculosis
HIV-TB	Human immunodeficiency virus–tuberculosis
IL	Interleukin
IFN-γ	Interferon gamma
TNF-α	Tumor necrosis factor alpha
CD	Cluster of differentiation
HL-DR	Human leukocyte antigen–DR isotype
PLHIV	People living with human immunodeficiency virus
ART	Antiretroviral therapy
Th1	T-helper 1
MTB	Mycobacterium tuberculosis
MHC	Major histocompatibility complex
TGF-β	Transforming growth factor-beta
TB-IRIS	TB-associated immune reconstitution inflammatory syndrome
Gp120	Glycoprotein 120
CCR5	C-C chemokine receptor 5
CXCR4	C-X-C motif chemokine receptor 4
DNA	Deoxyribonucleic acid
AICD	Activation-induced cell death
APC	Antigen presenting cells
ICOS	Inducible T cell Costimulator
PD-1	Programmed cell death protein
CTLA	Cytotoxic T-lymphocyte-associated antigen 4
LAG-3	Lymphocyte activation gene 3
TIM-3	T cell immunoglobulin and mucin-domain-containing protein 3
PD-L1/L2	Programmed cell death ligand ½
SHP1/P2	Scr homology region 2 (SH2) domain-containing phosphatase 1/2
ZAP-70	Zeta-chain-associated protein kinase 70
PLC-γ1	Phospholipase C-gamma 1
PI3K–AKT	Phosphatidylinositol 3-kinase-protein kinase B
PKCθ	Protein kinase C theta
mTOR	Mammalian target of rapamycin
RasMAPK/ERK	Rat sarcoma viral oncogene–mitogen-activated protein kinase–extracellular signal-regulated kinase
TIGIT	T cell immunoreceptor with immunoglobulin and immunoreceptor tyrosine-based inhibitory motif domains
NF-κB	Nuclear factor-kB
KLRG1	Killer cell lectin-like receptor G1
ATBI	Active tuberculosis infection
MTBI	Mycobacterium tuberculosis infection
AECs	Airway epithelial cells
PRRs	Pattern recognition receptors
TLRs	Toll-like receptors
NLRs	NOD-like receptors
DCs	Dendritic cells
NK	Natural killer
LTBI	Latent tuberculosis infection
NFAT	Nuclear factor of activated T cells
TCR	T cell receptor
BATF	Basic leucine zipper ATF-like transcription factor
IRF4	Interferon regulatory factor 4
CTLs	Cytotoxic T lymphocytes
TCF	T cell factor
MAIT	Mucosal-associated invariant T cells
iNKT	Invariant natural killer T cells
IgE	Immunoglobulin E
CYP2E1	Cytochrome P450 2E1
ROS	Reactive oxygen species
pCD4	Peripheral CD4
Tregs	Regulatory T cells
IDO2	Indoleamine 2,3-dioxygenase 2
ADORA2A	Adenosine A2A receptor
LAIR1	Leukocyte-associated immunoglobulin-like receptor 1
TNFSF14	Tumor necrosis factor superfamily member 14
CRP	C-reactive protein
suPAR	Soluble urokinase plasminogen activator receptor
TST	Tuberculin skin test
IGRAs	Interferon -γ release assays
HDTs	Host-directed therapies
ECs	Elite controllers
